# Supplementary figures and images for: Low Molecular Weight Hyaluronic Acid Effect on Dental Pulp Stem Cells In Vitro
Source: Biomolecules. 2020 Dec 28;11(1):22. doi: 10.3390/biom11010022 (PMC7823925; doi:10.3390/biom11010022)

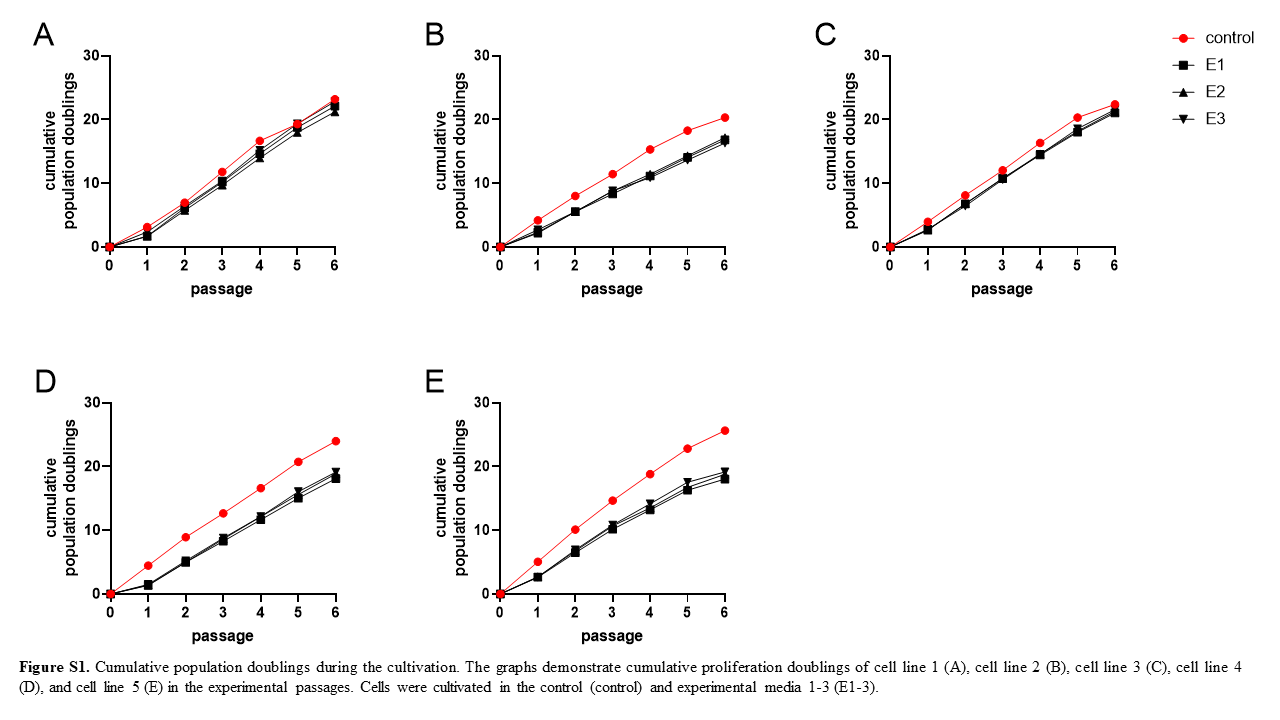

Supplement: Supplementary file 1 [file biomolecules-11-00022-s001.zip › Supplemetary/Figure_S1.tif]

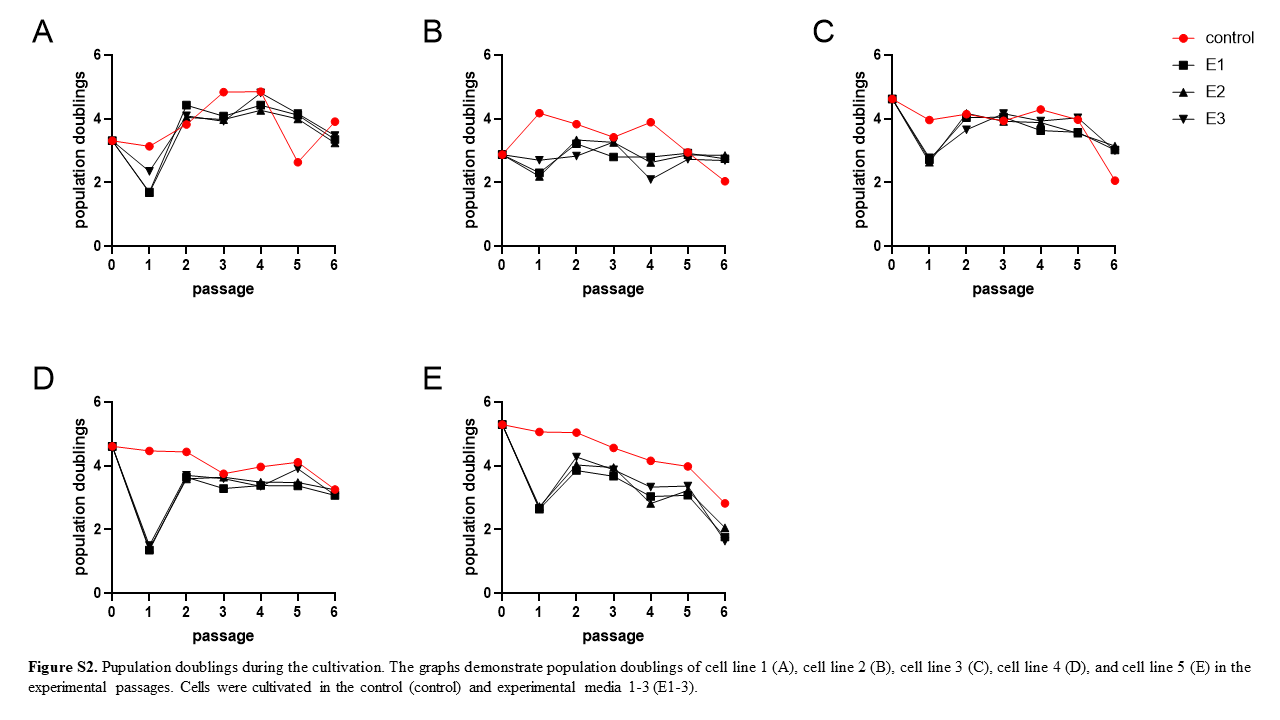

Supplement: Supplementary file 1 [file biomolecules-11-00022-s001.zip › Supplemetary/Figure_S2.tif]

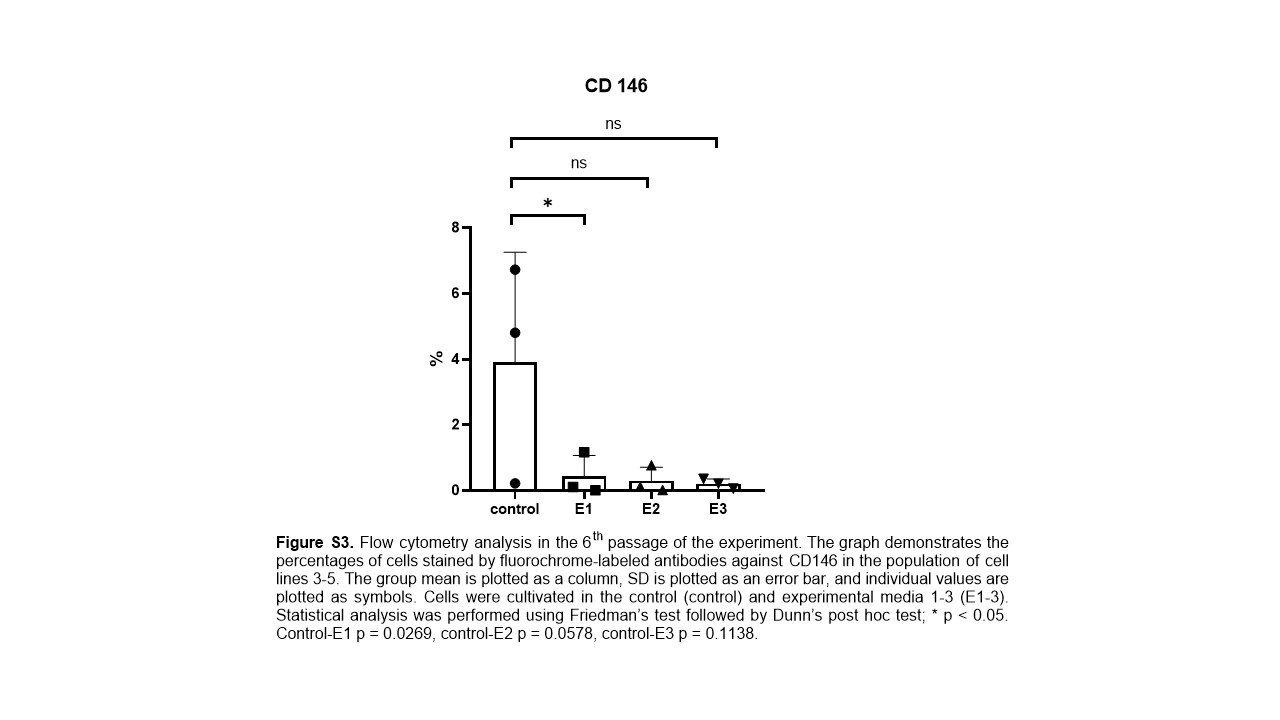

Supplement: Supplementary file 1 [file biomolecules-11-00022-s001.zip › Supplemetary/Figure_S3.tif]
